# A Patient With Blunt Trauma and Cardiac Arrest Arriving Pulseless at the Emergency Department; is that Enough Reason to Stop Resuscitation? Review of Literature and Case Report

**DOI:** 10.5812/ircmj.11623

**Published:** 2013-12-05

**Authors:** Alireza Hamidian Jahromi, Ashley Northcutt, Asser M Youssef

**Affiliations:** 1 Department of Surgery, Louisiana State University Health Sciences Center, Shreveport, Louisiana, USA

**Keywords:** Blunt trauma, Cardiac Arrest, Emergency Department, Thoracotomy, Blood transfusion, Laparotomy

## Abstract

The decision to stop or continue resuscitation in a patient with blunt trauma and cardiac arrest arriving pulseless to the hospital has always been controversial. While many authors still believe that it is a futile effort, with no chance of success for complete neurological recovery, some recent reports have challenged the idea. Here we report complete recovery of a severely injured patient following a motor vehicle accident who lost vital signs completely before arrival at our trauma center. No cardiac motion was detected on ultrasound examination on arrival. Emergency department thoracotomy, open cardiac massage, massive blood transfusion, damage control laparotomy with abdominal and pelvic packing, followed by angio-embolization of pelvic bleeding, and staged abdominal exploration were performed. This case is an example showing that resuscitation of patients with blunt trauma and cardiac arrest arriving pulseless to the hospital is not always futile.

## 1. Introduction

The decision to stop or continue resuscitation in a patient with blunt trauma and cardiac arrest arriving pulseless to the facility has always been controversial. Shimazu et al. (1983), in a report on 267 patients with cardiopulmonary arrest after trauma, mentioned that patients with blunt multisystem truncal injuries are unsalvageable (1). While many authors still believe resuscitation in these patients as a futile effort, with no chance of success for complete neurological recovery (2-5), some recent reports have challenged this idea, and successful resuscitations even with complete recovery have been reported (6-8).

Here we present a case of severely injured blunt trauma who arrived pulseless at our trauma center (Louisiana State University Health Sciences Center-Shreveport, The United States) in November 2011. No cardiac motion was detected on ultrasound examination. Early massive blood transfusion, emergency department thoracotomy (EDT), open cardiac massage, and a damage control laparotomy with abdominal and pelvic packing followed by angio-embolization of pelvic bleeding were performed, which gave the patient a chance to survive. The patient made a complete neurological recovery.

## 2. Case Report

A 23-year-old female involved in a motor vehicle collision was airlifted to our center. The initial Glasgow Coma Scale score was eight at the scene. Our trauma team which is led by an experienced trauma surgeon includes emergency room (ER) attending, 2 ER residents, 3 general surgery residents including the chief resident of surgery, anesthesiology senior resident, orthopedic resident, radiology resident, experienced ER nurses, and the X-ray technicians.

**Figure 1.**
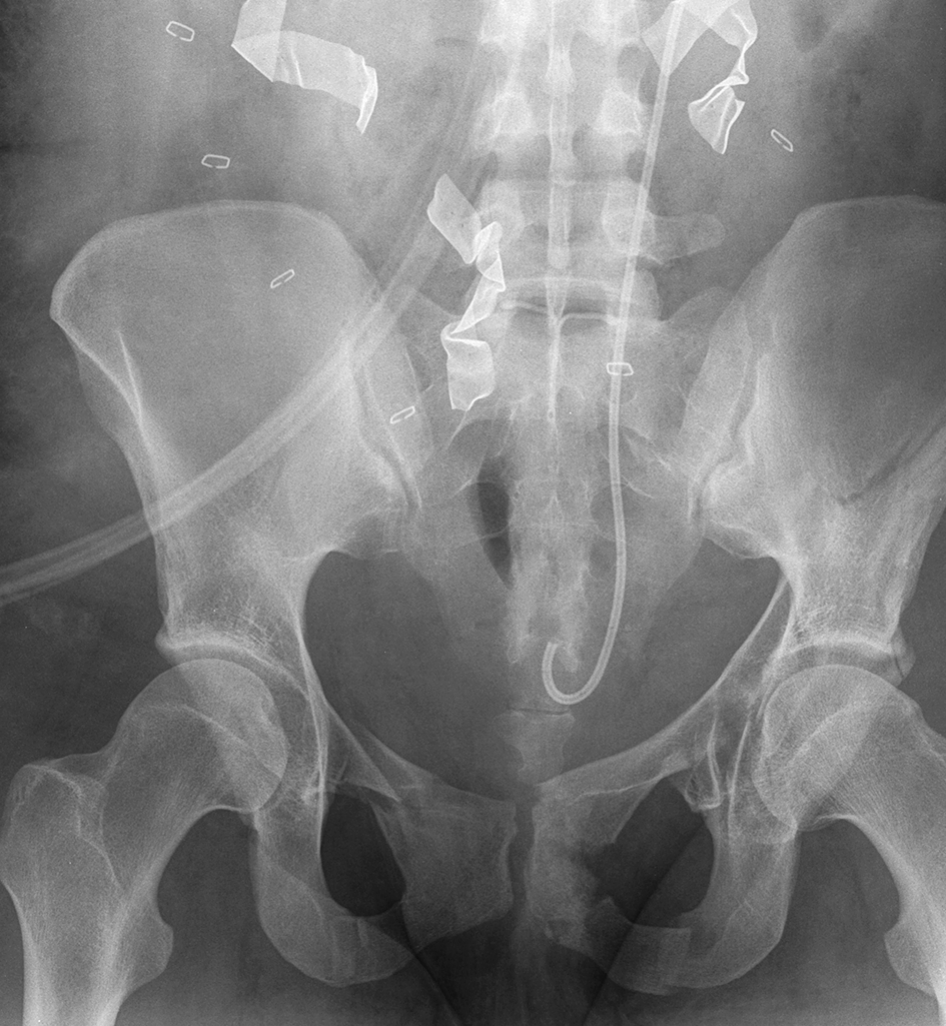
Plain pelvic x-ray (AP view) of the patient on admission showing extensive pelvic fractures

**Figure 2.**
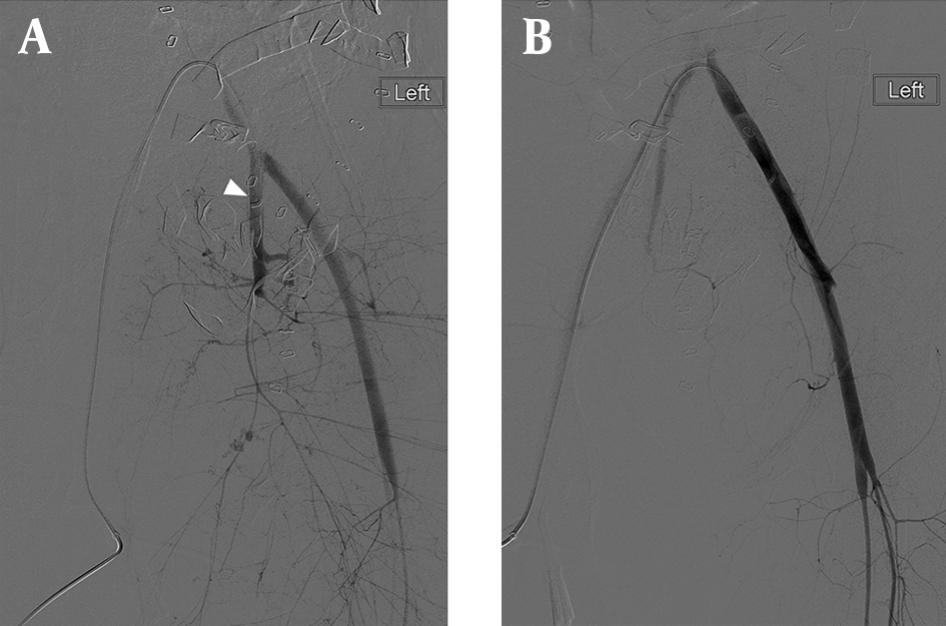
(A) Digital Subtraction Angiographic image of the left common iliac artery before (A) and after (B) angio-embolization of the left internal iliac artery (white arrowhead) which was performed to stabilize multiple pelvic bleeding sites

Upon arrival to the Emergency Department she had completely lost her vital signs. The Focused Assessment of Sonography for Trauma (FAST) examination revealed no cardiac motion and fluid in the abdomen. In the first 2 minutes after arrival, EDT was performed. There was very little blood in the heart, and no active source of bleeding was found in the chest. Direct cardiac massage was started followed by initiation of the massive transfusion protocol. Three units of packed red blood cells (PRBCs) were given rapidly during the Emergency Department admission period. Vital signs returned after a brief period (about 2 minutes) of direct cardiac massage. She had obvious, open pelvic fractures (Figure 1) with a bleeding perineal wound. The bleeding was controlled by external packing with gauze. The patient was then taken to the operating room (within 15 minutes of arrival), where a damage control laparotomy was performed. Upon entering the abdomen, a large amount of blood was noted, and an expanding pelvic hematoma was packed. The chest and abdomen were left open and a negative pressure dressing was applied. Resuscitation continued in the operating room with six units of warmed PRBCs, four units fresh frozen plasma (FFP), one unit of apheresis platelets, three liters of crystalloid, and two liters of albumin. The patient was taken to the intensive care unit (ICU) for continued resuscitation and rewarming. The patient’s initial temperature in the ICU was 94.6 F with a serum lactate of 5.7 mmol/L. In the ICU, 27 units of PRBCs, 18 units of FFP, and two units of cryoprecipitate were administered in a resuscitative fashion. The patient also received a dose of factor 7. She was on three pressors (levophed, dobutamine, and vasopressin). She received 500 cc of albumin and 300 cc of crystalloid during this time at ICU. When she was more stable, she was taken to the Interventional Radiology (IR) suite and underwent angio-embolization of the left internal iliac artery to stabilize the pelvic bleeding (Figure 2 A, 2B). On head CT-scan, she was noted to have an intraparenchymal hemorrhage, and an intracranial pressure (ICP) monitor was placed, which measured values in the 13-15 mmHg range. Once stabilized, patient returned to the operating room for re-exploration. The chest was closed. The abdomen was explored and the pelvic hematoma was found to be smaller and stable. Clots over the spleen and a large splenic laceration with active bleeding were noted, and a splenectomy was performed. The left ovary was found to be ischemic and was removed. An intraperitoneal bladder rupture was repaired. Negative pressure dressing was reapplied to the abdomen. Intraoperatively, one unit of PRBC, six units of FFP, and two units of packed platelets were given. Within the first 24 hours the patient received a total of 36 units of PRBCs, 28 units of FFP, three units of apheresis platelets, 3300 milliliters of crystalloid, 2500 milliliters of albumin, and two units of cryoprecipitate. Two days later the patient underwent abdominal closure. She was transferred to the floor after this operation. Following that, orthopedic surgeons repaired the left acetabular fracture. She has continued to do well and was discharged after 24 days with an intact neurological status. 

## 3. Discussion

The reported 0.25% survival rate of patients with blunt trauma after EDT in earlier reports would bring doubt into validity of this procedure (9). In fact some reports in the literature have questioned the role of EDT, specifically in the pediatric population (10, 11). Søreide reviewed the current literature and came to the conclusion that, although EDT is best used for isolated, penetrating injuries to the heart with reported survival rates of 31%, EDT would only give 1.6% chance of survival to patients with blunt trauma (12). Soreide also argued that according to the concept of damage control, which justifies lifesaving procedures at the price of (sometimes considerable) morbidities, the high rate of neurological deficiency in EDT survivors should not be considered as a rationale to prevent us from performing EDT in these situations (12).

A massive transfusion (MT) is defined as a transfusion greater than 10 units of blood products within a 24 hour period as a means of resuscitation for patients with trauma (13). MT has been described in relation to recent military experience, and has been proposed for civilian trauma (13). Multiple investigations of how to administer blood products in a damage control situation have been documented (14). Ratio-based transfusion protocols with higher ratios of FFP/PRBC and platelet/PRBC have helped to reduce the mortality and morbidity in the severely injured trauma cases requiring MT (15). Aggressive attainment of high transfusion ratios especially when started early during resuscitation, may reduce the transfusion requirement and may shift the overall blood requirements below those which currently define MT (15). In the current case, MT protocols using high ratios of FFP/PRBC and platelet/PRBC were implemented, while the patient was resuscitated. It has been shown that open cardiac massage is a more effective form of resuscitation in cardiac arrest than closed chest cardiac compression in blunt trauma, especially if the procedure is started within 20 minutes of the initial CPR attempt (16). Most patients with blunt trauma are severely injured with multiple organ systems affected; therefore, a resuscitative transfusion combined with the damage control approach plays a fundamental purpose in the survival of an exsanguinating trauma patient until hemostasis is obtained.

The timing of open cardiac massage is important in patients with hemorrhagic shock. Actively replacing the blood volume and redistributing blood circulation must be performed in concert until exsanguinations are controlled. Blunt trauma alone should not limit open cardiac resuscitation; however, the length of pulselessness is important (17). Expected survival rates from EDT decline precipitously the longer the time frame between open and closed chest compressions (18). The role of thoracotomy is well established in penetrating trauma; however, blunt trauma is less well known. An acceptable indication for thoracotomy is cardiac arrest in the trauma resuscitation unit or just prior to arrival (9). Only 3% of patients with severe blunt trauma present with hemorrhagic shock; however, they have a high mortality rate of about 60% (19). Tolerating permissive hypotension and resuscitating the patient with red blood cells and plasma allows for an earlier correction of the coagulation cascade (20). This in turn limits the amount of crystalloids; therefore, decreasing the effects of swelling on all organ systems. A disadvantage of massive blood transfusion is inflammatory reactions, such as lung injury. Due to the low incidence of acute lung injury in relation to the number of preventable deaths from hemorrhagic shock of patients with blunt trauma, it has been proven that early massive transfusion is beneficial (19).

In the current case, EDT with open cardiac massage started very early on after the patient’s arrival to our unit. Meanwhile, young age of this patient with an optimal health condition, an early start of massive blood transfusion protocol, along with a damage control laparotomy, abdominal and pelvic packing followed by angio-embolization of pelvic bleeding sites were the cornerstone of our success in keeping the patient alive with no neurologic morbidities. This case is an example showing that the resuscitation of patients with blunt trauma and cardiac arrest arriving pulseless to the hospital is not always futile.
